# Risk of first-time major cardiovascular event among individuals with newly diagnosed type 2 diabetes: data from Danish registers

**DOI:** 10.1007/s00125-023-05977-6

**Published:** 2023-08-02

**Authors:** Alexander C. Falkentoft, Thomas Alexander Gerds, Bochra Zareini, Filip K. Knop, Lars Køber, Christian Torp-Pedersen, Morten Schou, Niels E. Bruun, Anne-Christine Ruwald

**Affiliations:** 1 Department of Cardiology, Zealand University Hospital, University of Copenhagen, Roskilde, Denmark; 2 Department of Cardiology, Herlev-Gentofte Hospital, University of Copenhagen, Hellerup, Denmark; 3 Section of Biostatistics, Department of Public Health, University of Copenhagen, Copenhagen, Denmark; 4 Department of Cardiology, Nordsjællands Hospital, Hillerød, Denmark; 5 Center for Clinical Metabolic Research, Gentofte Hospital, University of Copenhagen, Hellerup, Denmark; 6 Department of Clinical Medicine, Faculty of Health and Medical Sciences, University of Copenhagen, Copenhagen, Denmark; 7 Novo Nordisk Foundation Center for Basic Metabolic Research, Faculty of Health and Medical Sciences, University of Copenhagen, Copenhagen, Denmark; 8 Steno Diabetes Center Copenhagen, Herlev, Denmark; 9 Department of Cardiology, Rigshospitalet, University of Copenhagen, Copenhagen, Denmark; 10 Department of Cardiology, Aalborg University Hospital, Aalborg, Denmark; 11 Clinical Institute, Aalborg University, Aalborg, Denmark

**Keywords:** Cardiovascular disease, Glucose-lowering drug, Glycaemic control, Remission of type 2 diabetes, Renin–angiotensin system inhibitor, Statin, Type 2 diabetes

## Abstract

**Aims/hypothesis:**

We aimed to examine whether individuals with initial omission of glucose-lowering drug treatment (GLDT), including those achieving initial remission of type 2 diabetes, may experience a higher risk of major adverse cardiovascular events (MACE) compared with well-controlled individuals on GLDT after a new type 2 diabetes diagnosis in real-world clinical practice. Furthermore, we examined whether a higher risk could be related to lower initiation of statins and renin–angiotensin system inhibitors (RASi).

**Methods:**

In this cohort study, we used Danish registers to identify individuals with a first measured HbA_1c_ between 48 and 57 mmol/mol (6.5–7.4%) from 2014 to 2020. Six months later, we divided participants into four groups according to GLDT and achieved HbA_1c_ (<48 vs ≥48 mmol/mol [6.5%]): well-controlled and poorly controlled on GLDT; remission and persistent type 2 diabetes not on GLDT. We reported how much the standardised 5 year risk of MACE could be reduced for each group if initiation of statins and RASi was the same as in the well-controlled group on GLDT.

**Results:**

We included 14,221 individuals. Compared with well-controlled participants on GLDT, the 5 year standardised risk of MACE was higher in the three other exposure groups: by 3.3% (95% CI 1.6, 5.1) in the persistent type 2 diabetes group not on GLDT; 2.0% (95% CI 0.4, 3.7) in the remission group not on GLDT; and 3.5% (95% CI 1.3, 5.7) in the poorly controlled group on GLDT. Fewer individuals not on GLDT initiated statins and RASi compared with individuals on GLDT. If initiation of statins and RASi had been the same as in the well-controlled group on GLDT, participants not on GLDT could have reduced their risk of MACE by 2.1% (95% CI 1.2, 2.9) in the persistent type 2 diabetes group and by 1.1% (95% CI 0.4, 1.9) in the remission group.

**Conclusions/interpretation:**

Compared with well-controlled individuals on GLDT, individuals not on initial GLDT had a higher 5 year risk of MACE, even among those achieving remission of type 2 diabetes. This may be related to lower use of statins and RASi.

**Graphical Abstract:**

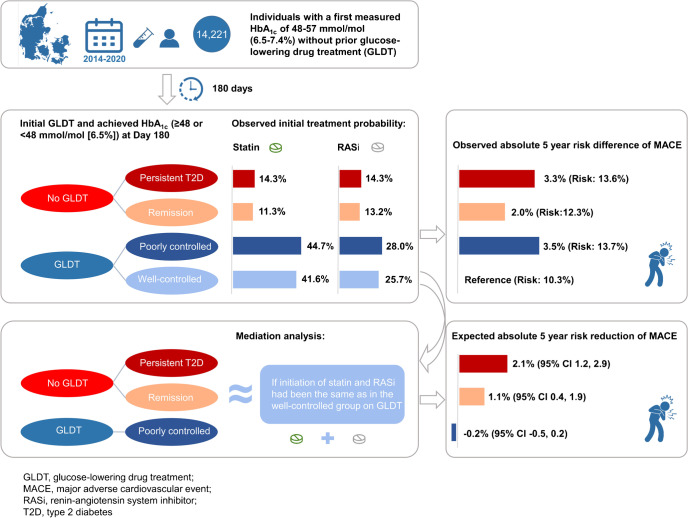

**Supplementary Information:**

The online version of this article (10.1007/s00125-023-05977-6) contains peer-reviewed but unedited supplementary material.


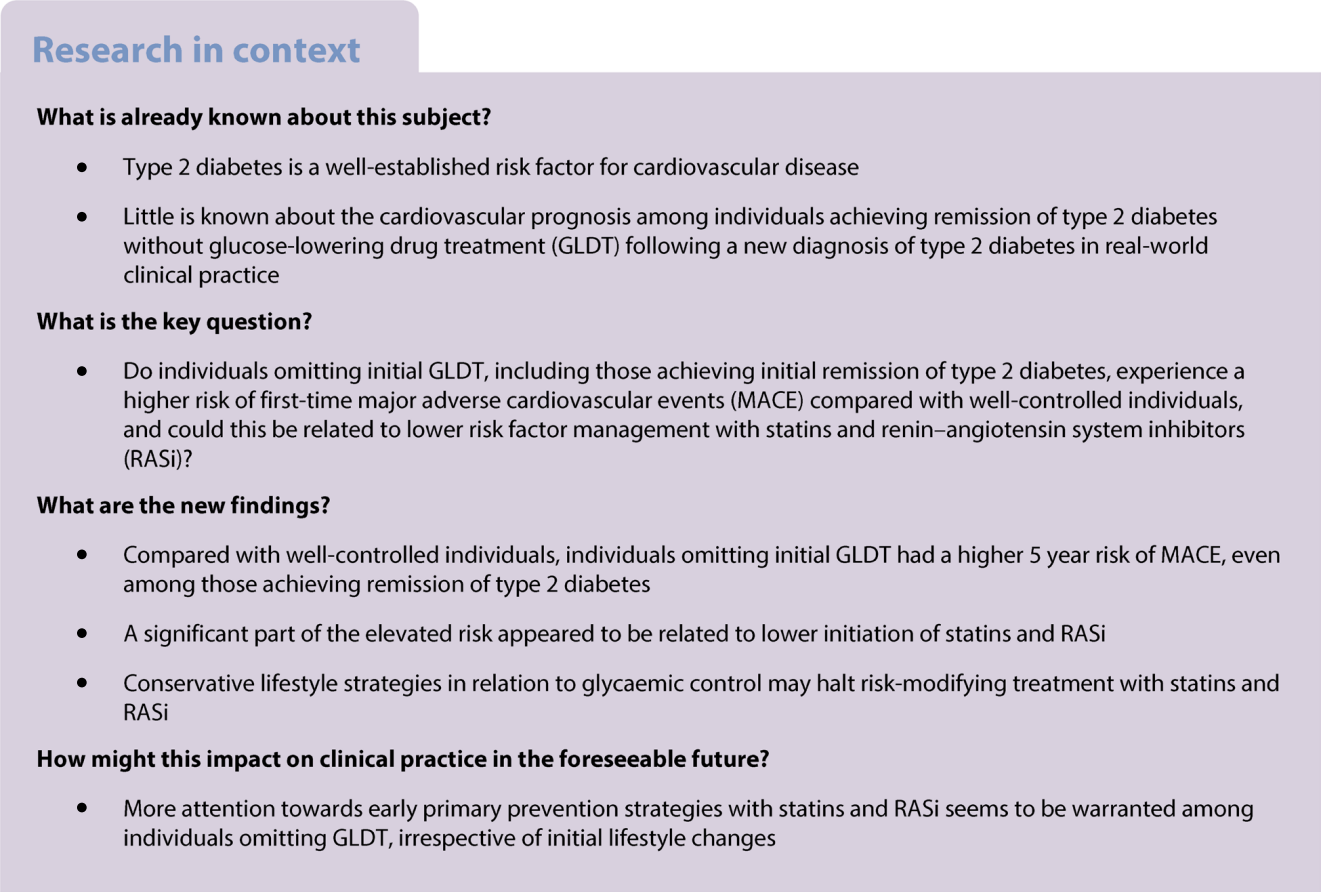


## Introduction

Type 2 diabetes is a progressive disease with an increased risk of cardiovascular morbidity [1]. Yet, little is known about the relationship between remission of type 2 diabetes and risk of cardiovascular events in real-world clinical practice. One English observational study found that remission was associated with a reduction of cardiovascular events among individuals with prevalent type 2 diabetes registered by primary care codes [2]. However, the likelihood of initial remission of type 2 diabetes and the associated risk of cardiovascular events remains to be examined among newly diagnosed individuals identified from routine HbA_1c_ measurements (≥48 mmol/mol [6.5%]). In accordance with guidelines presented by the ADA and the EASD [3], Danish guidelines allow a 6 month trial period of lifestyle modifications (diet and physical activity) prior to initiation of glucose-lowering drug treatment (GLDT) for individuals with an initial HbA_1c_ level below 58 mmol/mol (<7.5%) [4, 5]. These recommendations combined with the access to routine HbA_1c_ measurements from Danish registers provide a unique setting to investigate the influence of a possible initial conservative lifestyle strategy without GLDT.

A diagnosis of type 2 diabetes gives rise to new treatment targets of LDL-cholesterol and blood pressure, preferably with renin–angiotensin system inhibitors (RASi) [4–6]. Nonetheless, an initial conservative trial period of lifestyle modifications to achieve glycaemic control could lead to less aggressive risk factor management with statins and RASi. Thus, in a real-world usual care setting, individuals with remission or GLDT delay could have a higher risk of cardiovascular events compared with individuals initiating early effective GLDT. To reflect Danish guidelines, we examined the extent of GLDT delay and type 2 diabetes remission 6 months after a first measured HbA_1c_ between 48 and 57 mmol/mol (6.5–7.4%). Further, we examined whether these individuals may experience a higher risk of first-time cardiovascular events. As a secondary objective, we aimed to examine whether a higher risk could be mediated by lower initiation of statins and RASi. Hence, we aimed to test whether a reduction in risk of major adverse cardiovascular events (MACE) could be expected among individuals omitting initial GLDT if they had been as likely to receive initial treatment with statins and RASi as well-controlled individuals on GLDT (i.e. an indirect interventional effect).

## Methods

### Setting

In Denmark, the majority of patients with type 2 diabetes are managed in primary care. Since the WHO endorsed HbA_1c_ in the diagnostic criteria of diabetes (≥48 mmol/mol [6.5%]) in 2011 [7], HbA_1c_ measurements have been the main diagnostic method in Denmark [8].

### Data sources

All Danish residents are assigned a unique personal civil registration number, which allows cross-linkage of routinely collected individual-level data from the Danish nationwide administrative registers. In this cohort study, we obtained data from the following registers: (1) the Danish Civil Registration System, which holds data on date of birth, sex, ethnicity, migration, vital status and cohabitation status for all individuals residing in Denmark [9]; (2) the Danish National Patient Register [10]; (3) the Danish National Prescription Register, which holds data on redeemed prescriptions [11]; (4) the Danish Income Statistics Register [12]; (5) the Danish Population Education Register [13]. All these registers have previously been described [14]. Further, we also obtained information from the Danish Nationwide Register of Laboratory Results for Research, which holds systematic laboratory measurements from general practitioners, outpatient clinics and hospitalised patients in four out of five administrative regions in Denmark from 2014 onwards, coded according to the Nomenclature for Properties and Units (NPU) codes and local analysis numbers [15].

### Study population

**Fig. 1:**
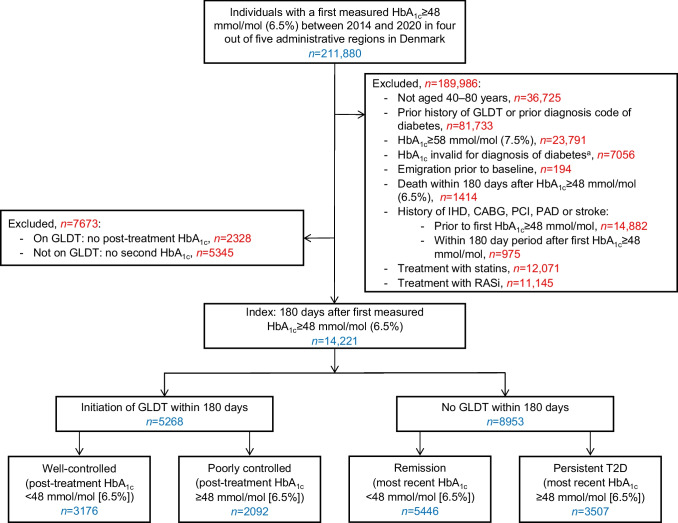
Flow chart of the study population. ^a^End-stage renal disease or eGFR<30 ml/min per 1.73 m^2^: *n*=1035; severe liver disease or splenomegaly: *n*=296; haemolytic anaemia, haemoglobinopathies, moderate anaemia, iron deficiency or B_12_ deficiency: *n*=3671; alcoholism: *n*=1362; severe hypertriglyceridaemia or severe hyperbilirubinaemia: *n*=49; and intake of specific drugs: *n*=643. CABG, coronary artery bypass graft surgery; IHD, ischaemic heart disease; PAD, peripheral artery disease; PCI, percutaneous coronary intervention; T2D, type 2 diabetes

We identified Danish residents who had a first-time HbA_1c_ measurement ≥48 mmol/mol (6.5%), from 1 January 2014 to 31 December 2020. We included individuals aged 40 to 80 years at the date of first measurement of HbA_1c_≥48 mmol/mol. Of these individuals, we included only those presenting with an HbA_1c_ level of 48–57 mmol/mol (6.5–7.4%). We excluded individuals with a prior redeemed prescription of any glucose-lowering drug (Anatomical Therapeutic Chemical [ATC] code: A10; https://www.whocc.no/atc_ddd_index/) or a prior primary discharge code of diabetes (ICD-8 codes: 249–250; ICD-10 codes: E10–E14, O24.0–O24.3, O24.5–O24.9 or H36.0; http://apps.who.int/classifications/icd10/browse/2016/en). Further, we excluded individuals with conditions in which HbA_1c_ is not appropriate for diagnosis of diabetes, including eGFR <30 ml/min per 1.73 m^2^ and moderate anaemia (haemoglobin <6.83 mmol/l) (see Fig. 1 for other criteria and electronic supplementary material [ESM] Tables 1, 2 for definitions). Moreover, we excluded individuals already on statins or RASi within 180 days prior to first measured HbA_1c_≥48 mmol/mol (6.5%).

In order to reflect the Danish recommendation of a possible 6 month trial period of lifestyle modifications [4, 5], we started the follow-up time for cardiovascular events 180 days after date of first measured HbA_1c_≥48 mmol/mol (6.5%) (i.e. the index date). As we aimed to investigate primary prevention outcomes, we included only individuals without prior registered ischaemic heart disease, stroke or peripheral artery disease at the index date. Individuals who did not initiate GLDT prior to the index date were included only if they had a second HbA_1c_ measurement recorded prior to index, while individuals who initiated GLDT prior to the index date were included only if they had a post-GLDT HbA_1c_ measurement recorded prior to index.

### Exposure

Initiation of GLDT was defined as at least one redeemed prescription (ATC code: A10). At index (180 days after first measured HbA_1c_≥48 mmol/mol [6.5%]), we divided participants into four exposure groups according to initiation of GLDT and most recently measured HbA_1c_ level: (1) well-controlled (HbA_1c_<48 mmol/mol) on GLDT; (2) poorly controlled (HbA_1c_≥48 mmol/mol) on GLDT; (3) remission (HbA_1c_<48 mmol/mol) not on GLDT; (4) persistent type 2 diabetes (HbA_1c_≥48 mmol/mol) not on GLDT. For individuals initiating GLDT, we only used HbA_1c_ measurements obtained after initiation of GLDT.

### Outcomes

The primary outcome was first-time MACE, defined as the first occurrence of myocardial infarction, stroke or all-cause death (see ESM Table 1 for ICD-10 codes). The diagnoses for myocardial infarction and stroke have a high validity (positive predictive values: myocardial infarction, 92.4–100%; stroke, 80.5–97.0%) [10, 16]. We examined initiation of statins and RASi in relation to exposure and MACE as described in the Statistical analyses section. Initiation of statins and RASi was defined as at least one redeemed prescription within the exposure window between the first measured HbA_1c_≥48 mmol/mol (6.5%) and the index date.

### Characteristics of the study population

Baseline medication was defined as a redeemed prescription within the period of 180 days prior to index date (see ESM Table 2 for ATC codes). Comorbidities were defined from discharge codes within the 10 years prior to index and/or by use of condition-specific medications (ESM Table 1). We obtained the most recent measurements of haemoglobin, LDL-cholesterol and creatinine within a 180 day period prior to date of first measured HbA_1c_≥48 mmol/mol (6.5%). eGFR was calculated from the Chronic Kidney Disease Epidemiology Collaboration (CKD-EPI) equation (see ESM Table 3 for NPU codes) [17].

### Statistical analyses

Patient characteristics at index date were summarised according to the four exposure groups, where continuous variables were presented as medians with IQRs and categorical variables as counts with percentages. We described the time-dynamic distribution of the individuals across exposure groups every 30 days during the 180 day period between the date of first measured HbA_1c_≥48 mmol/mol (6.5%) and the index date. We reported the proportions of the exposure groups who initiated statins and RASi treatment, respectively, before the index date. In addition, we reported the achieved post-index 1 year level of LDL-cholesterol according to the four exposure groups.

All participants were followed from the index date until date of MACE, emigration or end of study (31 January 2022), whichever came first. We used the Aalen–Johansen method to estimate the proportions of participants who had initiated statins and RASi, respectively, within 1 year after index, while accounting for MACE as a competing risk [18]. We used the stratified Kaplan–Meier method to estimate the crude 5 year absolute risks of MACE in the four exposure groups.

First, we aimed to examine the standardised absolute 5 year risk of MACE according to the four exposure groups using the observed within-group propensity of initiation of statins and RASi (total effect) [19]. Second, we aimed to estimate the indirect stochastic interventional effect (mediation effect) through initiation of statins and RASi in the three other exposure groups by setting the use of statins and RASi according to the observed propensity in the well-controlled group on GLDT. This method is similar to the methods suggested by Vanderweele et al and Rudolph et al [20, 21]. A minor variation is that we standardised to the whole population, in order to obtain estimates with a clinically relevant interpretation. We used logistic regression to model the propensity of initiation of statins and RASi, respectively, until the index date, separately in each exposure group. We used Cox regression to model the hazard rate of MACE in all participants according to exposure groups. In the Cox regression model, to avoid generalising the effects from other exposure groups, we allowed the effects of statins and RASi to vary between exposure groups by including an exposure × mediator interaction term. We further assumed consistency, positivity, conditional exchangeability (no uncontrolled exposure–outcome confounding, mediator–outcome confounding, or exposure–mediator confounding) and no intermediate confounders [19]. All regression models were adjusted for: age (5 year categories), sex, cohabitation status, income, ethnicity (Danish or immigrants/descendants); type of requester of first HbA_1c_ measurement (general practitioner or other); value of first measured HbA_1c_ (48–52 mmol/mol [6.5–6.9%] or 53–57 mmol/mol [7.0–7.4%]); eGFR prior to first HbA_1c_≥48 mmol/mol (6.5%); and history of comorbidities (heart failure, atrial fibrillation, chronic pulmonary obstructive disease and cancer).

Based on the Cox regression model and the observed characteristics of each participant, by setting the exposure level to each of the four given exposure levels by turn for each participant and by random assignment of treatment values of statins and RASi according to the modelled propensities, we predicted the absolute 5 year risks of MACE according to each exposure level for all participants [22]. We repeated the random treatment assignment 200 times and averaged the predicted risk estimates, in order to report the standardised absolute 5 year risk of MACE. First, we estimated the standardised absolute 5 year risk of MACE according to exposure and the observed propensity of statins and RASi in each of the four exposure groups. Second, for each of the three other exposure groups than the well-controlled group on GLDT, we estimated the indirect interventional effect of initiation of statins and RASi as the average standardised absolute 5 year MACE risk differences between setting treatment of statins and RASi according to the observed within-group propensity and the propensity in the well-controlled group on GLDT. The 95% CIs were obtained as quantiles of 1000 bootstrap samples obtained by resampling from the population of all included individuals.

Further, we report the separate indirect interventional effects (mediation effects) of statins and RASi. We also repeated the analysis of indirect interventional effects of the combined use of statins and RASi after excluding individuals with hypertension prior to first measured HbA_1c_≥48 mmol/mol. We also repeated the analyses in subgroups of sex and age (40–64, 65–80 years) and for the total population, replacing 180 days as index with 365 days. We defined the level of statistical significance as 5%, conducted all analyses in R, version 4.0.3, and used the riskRegression R package [23, 24].

### Ethics

In Denmark, register-based studies do not require ethical approval or informed consent by law. Permission to use data has been granted by the Knowledge Centre on Data Protection Compliance–the Capital Region of Denmark (approval number: P-2019-191).

## Results

### Characteristics of the study population

The final study population comprised 14,221 individuals with a first measured HbA_1c_ of 48–57 mmol/mol (6.5–7.4%) (Fig. 1). The distribution of GLDT and most recent HbA_1c_ level every 30 days during the first 180 days after first HbA_1c_≥48 mmol/mol (6.5%) are shown in ESM Fig. 1. The proportion of individuals receiving GLDT stabilised after 120 days, while the proportion of individuals with a follow-up HbA_1c_ measurement increased steadily over time. Hence, the distribution of exposure groups was as follows after 180 days (date of index): well-controlled on GLDT: 22.3%; poorly controlled on GLDT: 14.7%; remission not on GLDT: 38.3%; and persistent type 2 diabetes not on GLDT: 24.7%. Baseline characteristics differed according to initial GLDT and initial glycaemic control (Table 1). Regardless of initial glycaemic control, individuals on GLDT were slightly younger and had higher levels of first measured HbA_1c_ and eGFR than individuals not on GLDT. Regardless of GLDT, well-controlled/remitted individuals were more likely to be female, had higher income and educational level, and were less likely to live alone compared with the respective poorly controlled/persistent type 2 diabetes group on/not on GLDT. Compared with other groups, the well-controlled group on GLDT had fewer comorbidities. The majority of individuals on GLDT received metformin. No differences in LDL-cholesterol were observed between exposure groups. A similar pattern of baseline characteristics was observed according to exposure groups defined after 365 days (ESM Table 4).

**Table 1:** Baseline characteristics according to initial GLDT and glycaemic control, 180 days after first measured HbA_1c_≥48 mmol/mol (6.5%)

Variable	Initial GLDT		No initial GLDT	
	Well-controlled (HbA_1c_<48 mmol/mol) (*n*=3176)	Poorly controlled (HbA_1c_≥48 mmol/mol) (*n*=2092)	Remission (HbA_1c_<48 mmol/mol) (*n*=5446)	Persistent T2D (HbA_1c_≥48 mmol/mol) (*n*=3507)
Age, median [IQR]	58 [51, 66]	58 [50, 66]	60 [52, 68]	60 [52, 69]
Male sex, *n* (%)	1659 (52.2)	1134 (54.2)	2741 (50.3)	1855 (52.9)
Living alone, *n* (%)	1092 (34.4)	825 (39.4)	1943 (35.7)	1372 (39.1)
Income group, *n* (%)^a^				
Lowest	784 (25.0)	562 (27.4)	1246 (23.2)	909 (26.3)
Second lowest	791 (25.2)	568 (27.7)	1292 (24.1)	848 (24.6)
Second highest	810 (25.8)	497 (24.3)	1352 (25.2)	840 (24.3)
Highest	752 (24.0)	421 (20.6)	1474 (27.5)	853 (24.7)
Unknown	39	44	82	57
Educational level, *n* (%)^a^				
Basic	1050 (34.2)	737 (36.6)	1660 (31.4)	1138 (33.6)
High school or vocational	1401 (45.7)	912 (45.3)	2395 (45.2)	1488 (43.9)
Higher	616 (20.1)	365 (18.1)	1240 (23.4)	765 (22.6)
Unknown	109	78	151	116
Ethnicity, *n* (%)				
Native Danish	2614 (82.3)	1762 (84.2)	4593 (84.3)	2890 (82.4)
Immigrants/Descendants	562 (17.7)	330 (15.8)	853 (15.7)	617 (17.6)
Requested by a GP, *n* (%)	2953 (93.0)	1860 (88.9)	4838 (88.8)	3012 (85.9)
First HbA_1c_, *n* (%)				
48–52 mmol/mol 6.5–6.9%	2182 (68.7)	1109 (53.0)	4955 (91.0)	2823 (80.5)
53–57 mmol/mol 7.0–7.4%	994 (31.3)	983 (47.0)	491 (9.0)	684 (19.5)
eGFR prior to first HbA_1c_, *n* (%)^a^				
≥90 ml/min per 1.73 m^2^	1643 (54.6)	1153 (58.4)	2579 (49.8)	1648 (49.9)
60–89 ml/min per 1.73 m^2^	1245 (41.4)	749 (37.9)	2286 (44.1)	1478 (44.7)
30–59 ml/min per 1.73 m^2^	119 (4.0)	74 (3.7)	314 (6.1)	178 (5.4)
Unknown	169	116	267	203
LDL-cholesterol (mmol/l) prior to first HbA_1c_, median [IQR]	3.2 [2.7, 3.8]	3.2 [2.7, 3.8]	3.2 [2.6, 3.8]	3.3 [2.7, 3.9]
LDL-cholesterol prior to first HbA_1c_, *n* (%)^a^				
0–2.5 mmol/l	444 (21.0)	314 (23.0)	814 (22.1)	467 (20.3)
≥2.6 mmol/l	1674 (79.0)	1050 (77.0)	2877 (77.9)	1839 (79.7)
Unknown	1058	728	1755	1201
Comorbidities, *n* (%)				
COPD/Asthma	427 (13.4)	334 (16.0)	785 (14.4)	508 (14.5)
Cancer	126 (4.0)	102 (4.9)	264 (4.8)	176 (5.0)
Atrial fibrillation	59 (1.9)	45 (2.2)	176 (3.2)	93 (2.7)
Heart failure	39 (1.2)	40 (1.9)	102 (1.9)	82 (2.3)
Hypertension	597 (18.8)	476 (22.8)	946 (17.4)	635 (18.1)
Pharmacotherapy, *n* (%)^b^				
Insulin	24 (0.8)	86 (4.1)	–	–
Metformin	3122 (98.3)	2019 (96.5)	–	–
DPP-4i	37 (1.2)	59 (2.8)	–	–
SU	21 (0.7)	46 (2.2)	–	–
SGLT-2i	37 (1.2)	38 (1.8)	–	–
GLP-1RA	60 (1.9)	34 (1.6)	–	–
Statins	1322 (41.6)	935 (44.7)	618 (11.3)	501 (14.3)
Antithrombotics	170 (5.4)	116 (5.5)	204 (3.7)	145 (4.1)
RASi	817 (25.7)	585 (28.0)	719 (13.2)	501 (14.3)
β-blockers	310 (9.8)	222 (10.6)	592 (10.9)	421 (12.0)
Thiazide	299 (9.4)	211 (10.1)	527 (9.7)	341 (9.7)
Ca channel blockers	431 (13.6)	278 (13.3)	721 (13.2)	450 (12.8)
Loop diuretics	202 (6.4)	181 (8.7)	374 (6.9)	268 (7.6)

^a^The percentages indicate the proportions among individuals with complete data

^b^The dashes indicate that none initiated GLDT in these groups

COPD, chronic obstructive pulmonary disease; DPP-4i, dipeptidyl peptidase-4 inhibitors; GLP-1RA, glucagon-like peptide-1 receptor agonists; GP, general practitioner; SGLT-2i, sodium–glucose cotransporter 2 inhibitors; SU, sulfonylureas T2D, type 2 diabetes

### Five year risk of MACE according to initial GLDT and glycaemic control

**Fig. 2:**
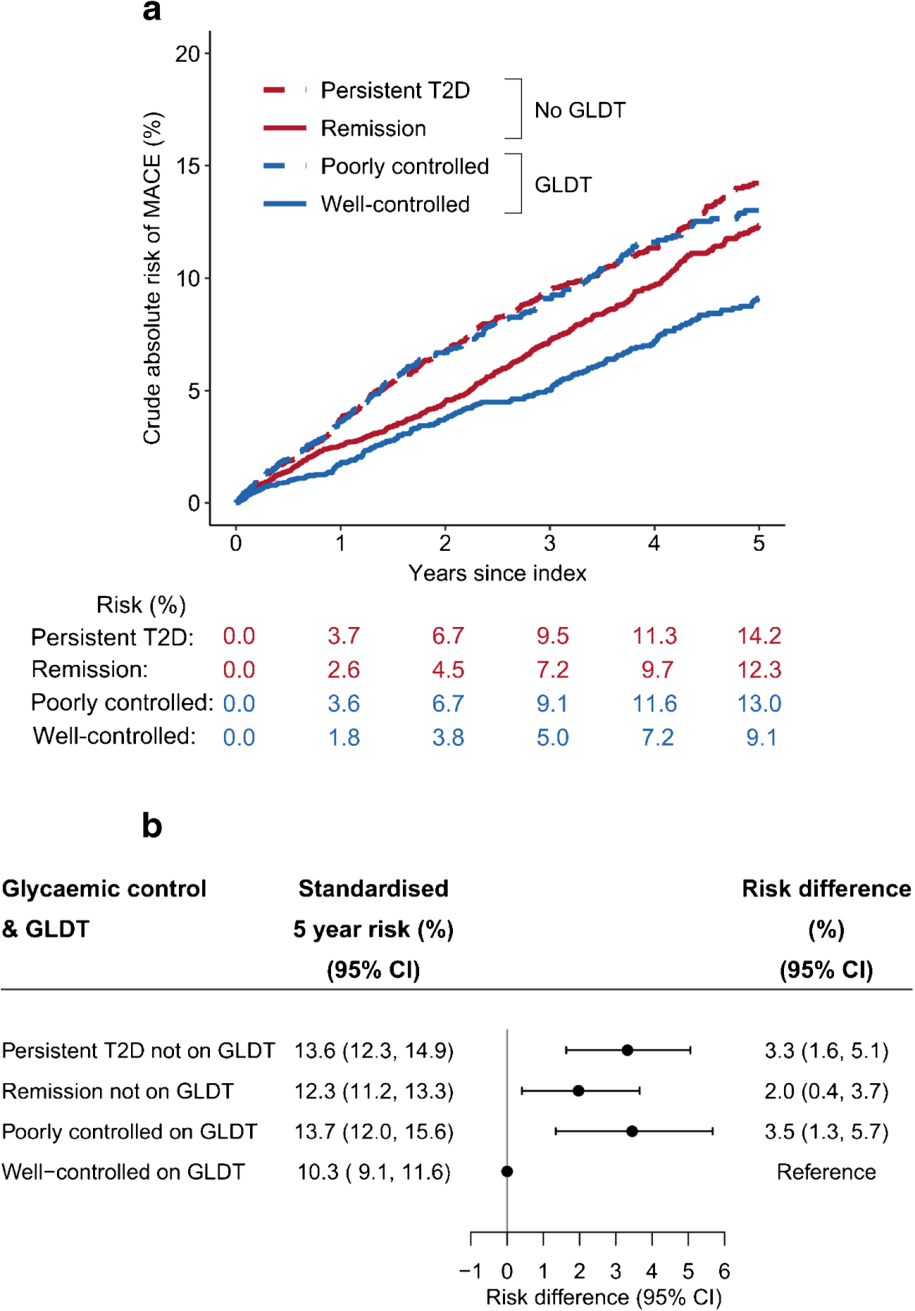
Crude (**a**) and standardised (**b**) absolute 5 year risk of first-time MACE according to initial GLDT and glycaemic control, 180 days after first measured HbA_1c_≥48 mmol/mol (6.5%). Time zero denotes 180 days after first measured HbA_1c_≥48 mmol/mol. Standardised to the distribution of all included individuals with respect to the distribution of age, sex, cohabitation status, ethnicity, income, type of requester (general practitioner or other), first measured HbA_1c_ level, eGFR, comorbidities, and after setting the use of statins and RASi as observed for each exposure group for all individuals. T2D, type 2 diabetes

During 52,006 person-years, 1351 individuals had a first-time MACE (stroke: 243, myocardial infarction: 161, all-cause death: 947). Well-controlled individuals on GLDT had the lowest unadjusted absolute 5 year risk of MACE compared with the three other exposure groups (Fig. 2a). After standardisation, the well-controlled group on GLDT remained with the lowest 5 year risk of MACE (Fig. 2b). Thus, compared with the well-controlled group on GLDT, the absolute standardised 5 year risk of MACE was 3.3% (95% CI 1.6, 5.1) higher in the group with persistent type 2 diabetes not on GLDT; 2.0% (95% CI 0.4, 3.7) higher in the remission group not on GLDT; and 3.5% (95% CI 1.3, 5.7) higher in the poorly controlled group on GLDT (Fig. 2b).

### Differences in initiation of statins and RASi according to initial GLDT and glycaemic control

**Fig. 3:**
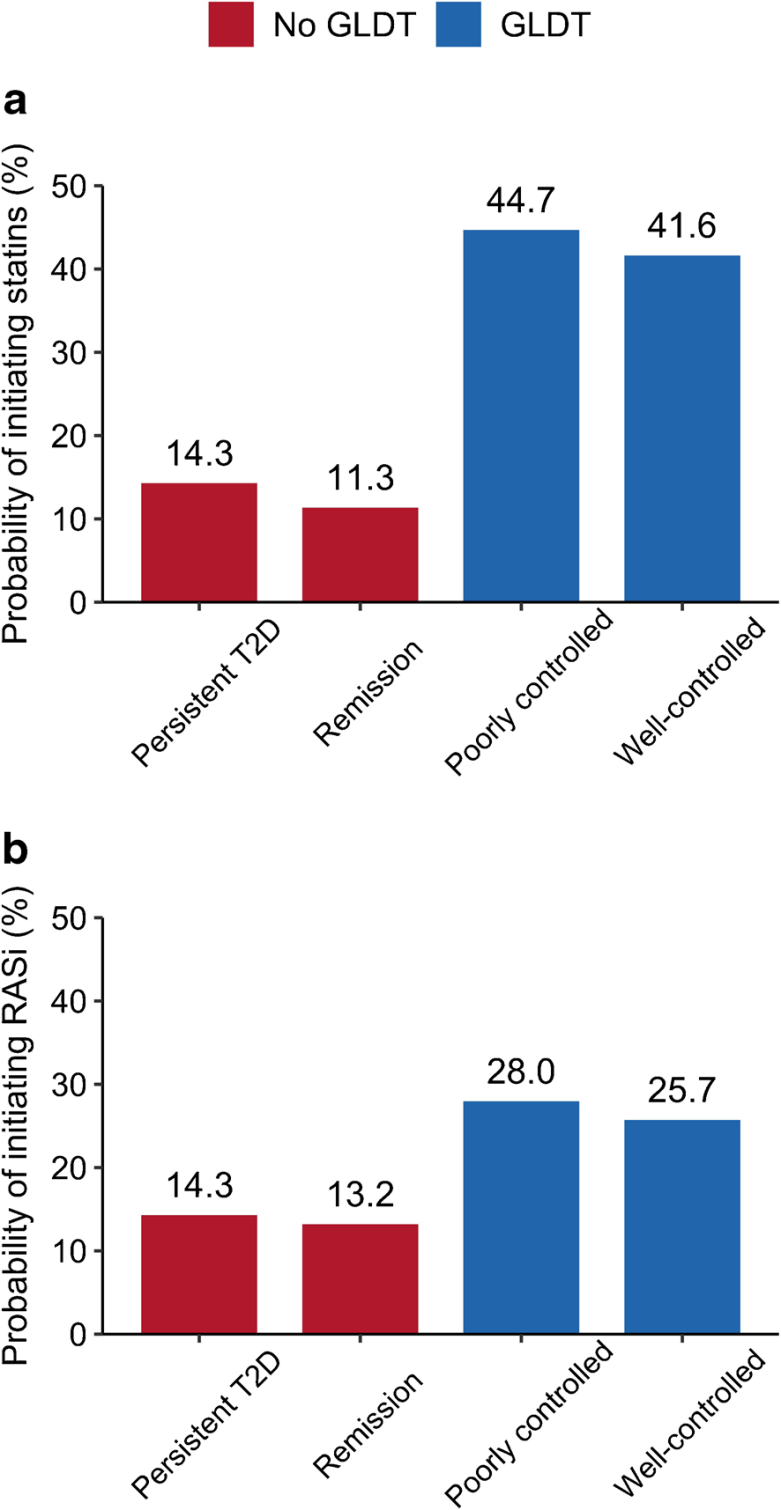
Probability of initiating statins (**a**) and RASi (**b**) according to GLDT and initial glycaemic control, 180 days after first measured HbA_1c_≥48 mmol/mol (6.5%). T2D, type 2 diabetes

Both groups omitting initial GLDT had lower probabilities of treatment with statins and RASi compared with individual on GLDT (Fig. 3). These differences were consistent in subgroups of age and sex (ESM Figs 2, 3). Moreover, the treatment differences with statins and RASi remained 1 year after index (ESM Table 5, ESM Fig. 4). The proportion of individuals achieving a 1 year LDL-cholesterol level below 1.8 mmol/l was around 26% among both groups on GLDT and around 11–12% among both groups not on GLDT (ESM Table 6). The proportion of individuals with missing follow-up LDL-cholesterol measurements was higher among individuals not on initial GLDT.

### Expected absolute reduction of 5 year risk of MACE if each exposure group had the same probability of receiving statins and RASi as the well-controlled group on GLDT

**Fig. 4:**
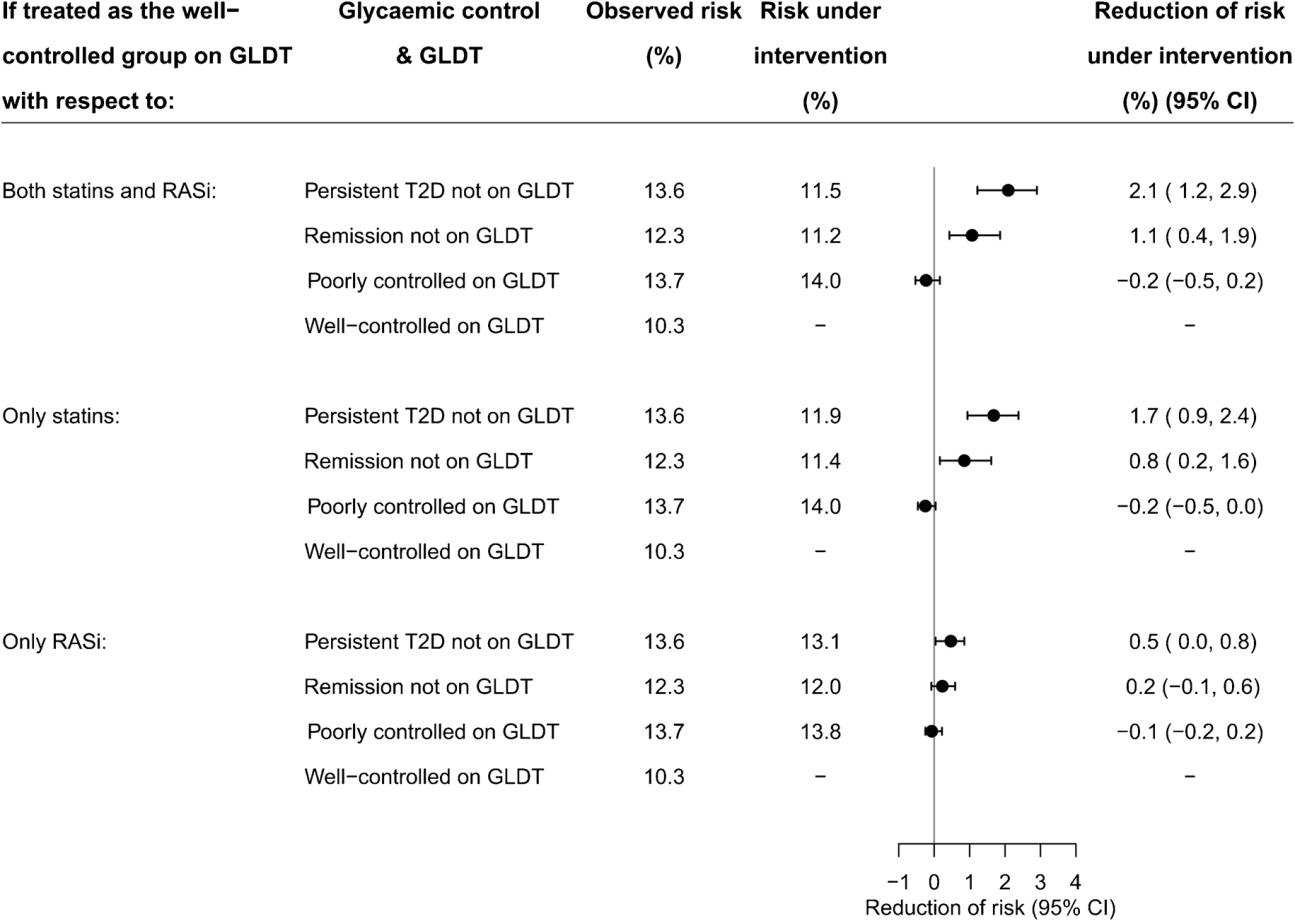
Expected absolute reduction of standardised 5 year risk of MACE if each exposure group had the same probability of receiving statins and/or RASi as the well-controlled group on GLDT. Depicted is the absolute reduction of standardised 5 year risk of MACE for each exposure group that could be expected if they, hypothetically, were as likely to receive treatment with statins and/or RASi as the well-controlled group on GLDT. The reduction of risk was calculated for each exposure group as the difference between the risk with the observed use of statins and/or RASi (observed risk) and the risk if treatment had been the same as the well-controlled group on GLDT (risk under intervention). The use of dashes for the well-controlled group on GLDT indicates that the observed risk and risk under intervention were identical for this specific group. T2D, type 2 diabetes

Figure 4 shows the absolute reduction of standardised risk of MACE that, hypothetically, could be expected for each exposure group if they had been as likely to receive initial treatment with statins and RASi as observed in the well-controlled group on GLDT. For both groups not on initial GLDT, this equalisation of initial treatment with statins and RASi could significantly reduce the standardised 5 year risk of MACE: by 2.1% (95% CI 1.2, 2.9) in the group with persistent type 2 diabetes and by 1.1% (95% CI 0.4, 1.9) in the remission group (Fig. 4). In separate analyses, equalising treatment with statins alone significantly changed the risk in both groups not on GLDT, whereas equalising treatment with RASi alone changed the risk significantly only in the group with persistent type 2 diabetes (Fig. 4). Of note, equalising treatment with both statins and RASi in the poorly controlled group on GLDT did not change the risk of MACE, as the observed probabilities of statins and RASi were almost identical as in the well-controlled group on GLDT (Fig. 4).

### Sensitivity analyses

Exclusion of individuals with hypertension prior to first measured HbA_1c_≥48 mmol/mol (6.5%) did not change our results (data not shown). Our results according to exposure were consistent across sex and age groups, yet with broad 95% CI limits (ESM Figs 2, 3, 5, 6). In sensitivity analyses, in which we defined the four exposure groups after 365 days instead of 180 days, the results were consistent with the main findings from this study (ESM Figs 7–9). Thus, regardless of achieved glycaemic control, individuals not on GLDT had lower probabilities of initiation of statins and RASi compared with individuals on GLDT (ESM Fig. 8). If the probabilities of treatment with statins and RASi had been the same as in the well-controlled group on GLDT, the excess absolute risk of 5 year MACE could be reduced by 2.6% (95% CI 1.6, 3.5) in the group with persistent type 2 diabetes not on GLDT and by 1.4% (95% CI 0.6, 2.1) in the remission group not on GLDT (ESM Fig. 9).

## Discussion

In a real-world setting of individuals with a first-time HbA_1c_ measurement above the diagnostic threshold of type 2 diabetes, we found a higher absolute 5 year risk of first-time MACE in individuals omitting initial GLDT as compared with well-controlled individuals on GLDT, regardless of having persistent type 2 diabetes or initial remission of type 2 diabetes. Further, individuals with initial omission of GLDT received markedly less treatment with statins and RASi. We showed that a significant part of the higher risk of MACE may be related to lower use of statins and RASi. These findings suggest that more attention could be given to risk-modifying treatment with statins and RASi among lifestyle-treated individuals without initial GLDT and underscore the disadvantages of a threshold-based, glucose-centred cardiovascular prevention strategy in real-world clinical practice.

One American and one English study found low rates of remission (2.8 and 9.7 per 1000 person-years) among individuals with prevalent type 2 diabetes [25, 26]. However, in support of our findings of type 2 diabetes remission as a common event after an initial type 2 diabetes diagnosis, they found relatively high remission rates among subgroups with low baseline HbA_1c_, no prior GLDT and short duration of type 2 diabetes [25, 26]. The evidence of a link between remission of type 2 diabetes and cardiovascular events is even more scarce. One study found an association between remission of type 2 diabetes and reduction of cardiovascular events [2]. These results were observed among individuals with prevalent type 2 diabetes identified from codes in primary care and are not generalisable to our study of new-onset type 2 diabetes identified from routine HbA_1c_ measurements. Moreover, the beneficial effects could be attributable to more intensive underlying lifestyle modifications than in our study or their choice of using the entire spectrum of type 2 diabetes as a reference group. Contrary to their study, we also examined whether individuals achieving remission could have had a better prognosis if they had been treated with statins and RASi as for individuals on GLDT. Thus, assuming that participants on GLDT were a heterogeneous group with regard to health behaviour, we also divided participants on GLDT into groups according to achieved HbA_1c_ and used the well-controlled group as a reference. Furthermore, we did not choose a very strict definition of remission, as we aimed to include spontaneous remission cases to reflect real-world decision making and the associated risk connected to undertreatment. This definition was almost in line with the recent consensus statement which defined remission as a single HbA_1c_ measurement below 48 mmol/mol (6.5%) 6 months after initiation of lifestyle modifications [27].

Remission may occur either spontaneously (i.e. weight loss due to illness or emotional distress, or by chance) or through lifestyle interventions [27]. As we aimed to include both spontaneous and intentional cases to reflect real-world clinical practice, lifestyle intervention studies may yield more favourable results than ours. Yet, only one randomised trial, the Look Action for Health in Diabetes (Look AHEAD) trial, has evaluated the effects of intensive lifestyle interventions (physical activity and diet) on cardiovascular outcomes [28]. The researchers did not report cardiovascular efficacy in overweight or obese individuals [28], in spite of increased remission rates [29]. The Diabetes Remission Clinical Trial (DiRECT) was not designed to evaluate cardiovascular outcomes, but showed sustained remission at 24 months for more than a third undergoing a very-low-calorie/energy diet [30]. Both DiRECT and the Look AHEAD trial reported lower use of cardiovascular medication in the intervention group [28, 30]. Consequently, although levels of systolic blood pressure and triglycerides were lower in the intervention group in both trials, total cholesterol or LDL-cholesterol levels were elevated and may have contributed to the lack of cardiovascular effects in the Look AHEAD trial [28, 30–32]. These results are consistent with prior evidence suggesting that weight reduction programmes typically show greater reductions in triglyceride levels with more modest reductions in total cholesterol and LDL-cholesterol [33]. Lifestyle interventions with sufficient intensity to prevent cardiovascular outcomes may only be achievable in a subset of individuals.

Our findings suggest that individuals with initial remission shortly after diagnosis remain at high risk. Identification of individuals at high cardiovascular risk is the cornerstone in prevention efforts, since the higher the absolute cardiovascular risk, the higher the absolute benefit of risk factor treatment [34]. Strong evidence shows that lowering of LDL-cholesterol reduces cardiovascular risk in many different types of individuals, regardless of presenting cholesterol level [35, 36]. Further, based on a recent large individual participant-level data meta-analysis, it has been suggested that initiation of antihypertensive treatment may be based on an individual’s risk rather than relying on blood pressure values [37]. Moreover, guidelines recommend inclusion of RASi in treatment of hypertension in type 2 diabetes, with strong evidence in the presence of albuminuria or proteinuria and some evidence in the absence of kidney disease [4–6, 38].

Our results indicated that an absolute risk reduction of MACE could be expected in both groups not on initial GLDT if they had been as likely to receive statins and RASi therapy as the well-controlled individuals on GLDT. The most prominent absolute risk reductions after equalisation of treatment were observed in the group with persistent type 2 diabetes (with the highest cardiovascular risk). As we allowed for exposure × mediator interaction in our Cox model, the slightly more conservative findings in the remission group could also in part arise from confounding by indication, as treated individuals may have had a higher prevalence of undetected cardiovascular risk factors such as smoking and blood pressure.

Another large Danish register-based cohort study found that an HbA_1c_ just below the diagnostic threshold (46–47 mmol/mol [6.4–6.5%]) predicts the highest risk of MACE in individuals with a first measured HbA_1c_ of 40–51 mmol/mol (5.8–6.8%) [39]. Further, these individuals received much less treatment with statins and RASi compared with individuals just above the threshold. Our study elaborates on these findings by also reporting an increased cardiovascular risk for individuals with remission after an initial HbA_1c_ measurement above the diagnostic threshold of type 2 diabetes. Moreover, our results suggest that the increased use of statins and RASi associated with the more aggressive treatment targets after a diagnosis of type 2 diabetes mostly seems to extend to individuals treated with GLDT [4–6]. Further, our findings that lower use of statins and RASi may be connected to a large part of the excess risk of MACE in individuals omitting initial GLDT are in line with previous findings in prediabetes, which have shown that elevated cardiovascular risk is mostly attributable to established risk factors other than dysglycaemia [40–42].

We sought to examine real-world initiation patterns and risk of cardiovascular outcomes with respect to guidelines for a possible 6 month lifestyle modification period without GLDT. Therefore, we aimed to report the risk of MACE according to whether individuals had received GLDT and their achieved HbA_1c_ level at a single time point, 180 days following their first diagnostic routine measurement of type 2 diabetes. Further, we examined whether differences in risk could be attributed to differences in initiation of statins and RASi. Notably, we did not aim to determine the effects of GLDT and glycaemic control, including the time-varying effects. Thus, we considered possible relapse of type 2 diabetes after initial remission as part of the causal path between initial remission and MACE in real-world clinical practice. Moreover, our findings with regard to glycaemic control should be carefully interpreted. Although our findings suggest that poorly controlled type 2 diabetes predicts higher cardiovascular risk, this could be attributed to a more severe variant of type 2 diabetes and/or less toleration of intensive GLDT.

### Clinical implications

We report potential mediation effects of statins and RASi on average group level and not treatment effects. Our findings are hypothesis generating and suggest that conservative strategies in relation to glycaemic control may halt risk-modifying treatment with statins and RASi after an initial diagnosis of type 2 diabetes. Given that the timing of pharmacologic treatment to achieve LDL-cholesterol (at least <2.6 mmol/l) and blood pressure (<130/<80 mmHg) targets has not been clearly specified in Danish and European diabetes guidelines, a discussion of timing seems relevant [4–6].

### Strengths and limitations

The major strengths of this study arise from the large amount of unselected individual data on routine HbA_1c_ measurements, prescriptions, sociodemographic variables and diagnosis codes with high validity. However, there are also limitations of which we need to be aware.

First, although we adjusted for initial levels of HbA_1c_ and eGFR and baseline comorbidities, we cannot eliminate the possibility of unmeasured confounding because we lacked information on several clinically important variables, including smoking, weight, diet, physical activity, duration of type 2 diabetes and blood pressure. In the two groups not on GLDT, unmeasured confounding may have led to a slight overestimation when setting the use of statins and RASi as observed in the well-controlled group on GLDT. However, as unmeasured confounders may be more often present in individuals on initial GLDT, unmeasured confounding may have biased the absolute risk estimates of MACE towards the null in the two groups not on GLDT. Likewise, we would expect unmeasured confounding by indication to bias the mediation effects of statins and RASi towards the null.

Second, we focused on initial treatment differences with statins and RASi. Yet, the differences in use of statins and RASi were maintained throughout follow-up and the restriction of initial treatment most likely contributed with estimates towards the null. Third, we cannot exclude that initiation of statins and RASi may also be surrogates for higher levels of health literacy and a more favourable underlying health, which might be linked to more intensive non-pharmacological interventions such as self-management education and lifestyle. As we allowed for exposure × mediator interaction, this may be especially present in the persistent type 2 diabetes group and could bias our results away from the null. However, confounding by indication would bias the results towards the null and a ‘healthy adherer effect’ was previously shown to be minimal compared with pharmacological drug use [43]. Fourth, we cannot determine whether the observed treatment differences were attributable to doctors not prescribing or patients not redeeming prescriptions. In addition, although we considered redeemed prescriptions as a valid proxy of treatment utilisation, we cannot exclude the possibility of misclassification. Regarding statins and RASi use, this misclassification may have been most apparent in the two groups omitting initial GLDT, leading to a possible bias towards the null. Fifth, although we would expect our findings to be more pronounced with longer follow-up, our findings obtained from relatively short follow-up may not be generalisable to long-term outcomes. Last, given the observational study design, our findings represent associations, and no causal conclusions can be drawn.

## Conclusionss

Following a first-time HbA_1c_ measurement above the diagnostic threshold of type 2 diabetes, a large group of individuals omitting initial GLDT had a higher absolute 5 year risk of first-time MACE compared with well-controlled individuals on GLDT. This was observed even for individuals achieving initial remission of type 2 diabetes without GLDT. A significant part of the excess risk of MACE appeared to be related to lower use of statins and RASi. This study supports the importance of considering early pharmacological primary prevention with statins and RASi in addition to lifestyle changes.

### Supplementary Information

Below is the link to the electronic supplementary material.

Supplementary file1 (PDF 1586 KB)

## Data Availability

The highly protected servers of Statistics Denmark hold the data used for this study. Danish data protection laws do not allow sharing of these data.

## Abbreviations

ATC: Anatomical Therapeutic Chemical
DiRECT: Diabetes Remission Clinical Trial
GLDT: Glucose-lowering drug treatment
Look AHEAD: Look Action for Health in Diabetes
MACE: Major adverse cardiovascular events
NPU: Nomenclature for Properties and Units
RASi: Renin-angiotensin system inhibitors

## Authors’ relationships and activities

FKK has served on scientific advisory panels and/or been part of speaker’s bureaus for, or served as a consultant to and/or received research support from 89bio, AstraZeneca, Bayer, Boehringer Ingelheim, Dawn Health, Eli Lilly, Gubra, Novo Nordisk, Pharmacosmos, Sanofi, Structure Therapeutics, Zealand Pharma and Zucara not related to the current study; and is a minority shareholder in Antag Therapeutics ApS and co-owner of Medicinsk Vægttab ApS. LK reports speaker’s fees from AstraZeneca, Bayer, Boehringer Ingelheim, Novartis and Novo Nordisk not related to the current study. CT-P has received grants for studies from Bayer and Novo Nordisk not related to the current study. MS reports speaker’s fees from Novo Nordisk, AstraZeneca, Novartis and Boehringer Ingelheim unrelated to the current work. NEB has received investigator-initiated grants from the Novo Nordisk Foundation, the Augustinus Foundation, the Kaj Hansen Foundation and Health Insurance Denmark unrelated to the current work. A-CR reports speaker’s fees from Novartis, AstraZeneca and BMS unrelated to the current study. The authors declare that there are no other relationships or activities that might bias, or be perceived to bias, their work.
