# Supplementary material for: Risk of first-time major cardiovascular event among individuals with newly diagnosed type 2 diabetes: data from Danish registers
Source: Diabetologia. 2023 Aug 2;66(11):2017–29. doi: 10.1007/s00125-023-05977-6 (PMC10541344; doi:10.1007/s00125-023-05977-6)
Supplement: Supplementary file 1 — Supplementary file1 (PDF 1586 KB) [file 125_2023_5977_MOESM1_ESM.pdf]

Electronic supplementary material

Risk of first-time major cardiovascular event among individuals with newly diagnosed type 2 diabetes: data from Danish registers

Authors: Alexander C. Falkentoft; Thomas Alexander Gerds; Bochra Zareini; Filip K. Knop; Lars Køber; Christian Torp-Pedersen; Morten Schou; Niels E. Bruun; Anne-Christine Ruwald

Table of content:

ESM Table 1. Definition of outcomes and comorbidities …………………………………………………...3

ESM Table 2. Definition of medication……………………..……………………………………….5

ESM Table 3. Laboratory measurements………………………..…………………………………..6

ESM Table 4. Baseline characteristics according to glucose-lowering drug treatment and glycaemic control, 365 days after first measured HbA1c ≥ 48 mmol/mol……………………………………………..7

ESM Table 5. Probabilities of initiating statins and RASi according to glucose-lowering drug treatment and initial glycaemic control, 365 days after index…..……………………………………………………9

ESM Table 6. Level of LDL cholesterol according to glucose-lowering drug treatment and initial glycaemic control, 365 days after index………………………………………………………………..10

ESM Figure 1. Distribution of glucose-lowering drug treatment and most recent HbA1c level at each time point up to 180 days after first measured HbA1c ≥ 48 mmol/mol……………………….……….11

ESM Figure 2. Probability of initiating statins and RASi according to glucose-lowering drug treatment and glycaemic control, 180 days after first measured HbA1c ≥ 48 mmol/mol, among men, stratified by age…………………………………………………………………………………………………..…12

ESM Figure 3. Probability of initiating statins and RASi according to glucose-lowering drug treatment and glycaemic control, 180 days after first measured HbA1c ≥ 48 mmol/mol, among women, stratified by age…..…………………………………………………………………………………………………13

ESM Figure 4. Probabilities of initiating statins and RASi within one year after index date according to glucose-lowering drug treatment and initial glycaemic control…..………………………………………14

ESM Figure 5. Standardised absolute 5 year risk of MACE according to glucose-lowering drug treatment and glycaemic control, 180 days after first measured HbA1c ≥ 48 mmol/mol, stratified by age and sex……………………………………………………………………………………………………….15

ESM Figure 6. Expected absolute reduction of standardised 5 year risk of MACE if each exposure group had the same probability of receiving statins and RASi as the well-controlled group on glucose-lowering drug treatment, stratified by sex and age……………….………………………………………………16

ESM Figure 7. Standardised absolute 5 year risk of first-time MACE according to initial glucose-lowering drug treatment and glycaemic control, 365 days after first measured HbA1c ≥ 48 mmol/mol……………………………………………………………………………………………….17

ESM Figure 8. Probability of initiating statins and RASi according to glucose-lowering drug treatment and initial glycaemic control, 365 days after first measured HbA1c ≥ 48 mmol/mol……………………..18

ESM Figure 9. Expected absolute reduction of standardised 5 year risk of MACE if each exposure group had the same probability of receiving statins and RASi as the well-controlled group on glucose-lowering drug treatment, 365 days after first measured HbA1c ≥ 48 mmol/mol………..………………………..19

ESM Table 1. Definition of outcomes and comorbidities

	Details	ICD-8, ICD-10 code, or NCSP	ATC code
**Outcomes of interest**	Identified by primary or secondary in-patient discharge diagnosis codes from the Danish National Patient Register.		
Stroke		ICD-10: I60-I61, I63-I64	
Myocardial infarction		ICD-10: I21+I22	
All-cause death		Any registered date of death	
**History of cardiovascular disease registered at any time prior to index**	Identified by primary or secondary in- or out-patient discharge diagnosis codes from the Danish National Patient Register.		
Ischemic heart disease:	Myocardial infarction:<br><br>Other ischemic heart disease:<br><br>Percutaneous coronary intervention:<br><br>Coronary artery bypass graft surgery:	ICD-10: I21 + I22<br><br>ICD-8: 410-414<br>ICD-10: I20, I23-I25<br><br>NCSP: KFNG<br><br>NCSP: KFNA, KFNB, KFNC, KFND, KFNE	
Peripheral artery disease		ICD-8: 443<br>ICD-10: I70, I739	
Stroke		ICD-8: 430-434, 436<br>ICD-10: I60-I61, I63-I64	
Heart failure		ICD-8: 425, 42709-42711, 428<br>ICD-10: I110, I130, I132, I420, I426-I429, I50	
**Other comorbidities registered within 10 years prior to baseline**	Identified by primary or secondary in- or out-patient discharge diagnosis codes from the Danish National Patient Register.		
Atrial fibrillation		ICD-10: I48	
Hypertension	Baseline hypertension was defined as either a diagnosis with hypertension or as redemption of at least two classes of antihypertensive drugs within 180 days prior to the index date, as previously validated.[3,4]	ICD-10: I110-I115	α adrenergic blockers (C02A, C02B, C02C), non-loop diuretics (C02DA, C02L, C03A, C03B, C03D, C03E, C03X, C07B, C07C, C07D, C08G, C09BA, C09DA, C09XA52), vasodilators (C02DB, C02DD, C02DG), β blockers (C07A, C07B,

			C07C, C07D, C07F), calcium channel blockers (C07F, C08, C09BB, C09DB), and renin-angiotensin system inhibitors (C09AA, C09BA, C09BB, C09CA, C09DA, C09DB, C09XA02, C09XA52)
Chronic obstructive pulmonary disease (COPD) or asthma	Baseline COPD or asthma was defined as either a diagnosis with COPD or at least one redemption of drugs for obstructive airway disease within 180 days prior to the index date.	ICD-10: J42, J44	Inhaled corticosteroids (R03BA, RO3AK06-R03AK12, R03AL08-R03AL09), inhaled short- and long-acting β2-agonists (R03AC, R03AK06-R03AK13, R03AL01-R03AL09), inhaled short- and long-acting muscarinic antagonists (R03BB, R03AL01-R03AL09)
End-stage renal disease		ICD-10: N185, Z992	
Cancer		ICD-10: C00-C97	
Alcoholism		ICD-10: E244, E529A, F10, G312, G621, I426, K70, K852, K860, L278A, T51, Z714, Z721,	
Severe liver disease/splenomegaly		ICD-10: B150, B160, B162, B190, I85, K703, K704, K717, K72, K743-K746, K766, I982, R16, D709E, K768B, Q890C, Z944	
B12 deficiency	Registered diagnoses code or registered hospital administered B12 supplementation 180 days prior to first measured HbA1c ≥ 48 mmol/mol	ICD-10: D51, E538D<br><br>Administration: BOHC2	
Iron deficiency	Registered diagnoses code or registered hospital administered iron supplementation 180 days prior to first measured HbA1c ≥ 48 mmol/mol	ICD-10: D50, E611<br><br>Administration: BOHC1	
Haemoglobinopathies/ haemolytic anaemia		ICD-10: D56-D59	

ICD, International Classification of Diseases; NCSP, Nordic Medico-Statistical Committee Classification of Surgical Procedures.

ESM Table 2. Definition of medication

**Pharmacotherapy**	**Details and ATC codes**
	Identified from the National Prescription Register.
**Glucose-lowering drug treatment**	A10
Metformin	A10BA02, A10BD07, A10BD08, A10BD10, A10BD11, A10BD13, A10BD15, A10BD16, A10BD20, A10BD23
SGLT-2i	A10BK, A10BD15, A10BD16, A10BD20, A10BD23
GLP-1RA	A10BJ
DPP-4i	A10BH, A10BD07, A10BD08, A10BD10, A10BD11, A10BD13
Sulfonylureas	A10BB
Insulin	A10A
**Cardiovascular medication**	
Statins	C10AA
Renin-angiotensin system inhibitors	C09
Antithrombotics	B01AC
Beta blockers	C07
Loop diuretics	C03C
Thiazide	C03A
Calcium channel blockers	C08
**Drugs for which HbA1c is invalid for diagnosis of diabetes**	
Trimethoprim	J01EA
Sulfamethoxazole	J01EE
Sulfasalazine	A07EC01
Hydroxyurea	L01XX05
Dapsone	J04BA02

ESM Table 3. Laboratory measurements.

**Blood samples**	**Details and codes (NPU codes and local analysis numbers)**
	Identified from the National Laboratory Database
HbA1c	NPU27300, NPU03835
Creatinine	NPU04998, NPU18016
LDL cholesterol	NPU01568, NPU10171, DNK35308
Triglycerides	NPU03620, NPU04094
Haemoglobin	NPU02319
Bilirubin	NPU01370

NPU, Nomenclature for Properties and Units.

ESM Table 4. Baseline characteristics according to glucose-lowering drug treatment and glycaemic control, 365 days after first measured HbA1c ≥ 48 mmol/mol.

Variable	Initial GLDT		No initial GLDT	
	Well-controlled (HbA1c < 48 mmol/mol) (*n*=4000)	Poorly controlled (HbA1c ≥ 48 mmol/mol) (*n*=2794)	Remission (HbA1c < 48 mmol/mol) (*n*=5561)	Persistent T2D (HbA1c ≥ 48 mmol/mol) (*n*=2896)
Age, median [IQR]	58 [51, 67]	57 [50, 65]	60 [52, 69]	60 [52, 69]
Male sex, *n* (%)	2082 (52.0)	1517 (54.3)	2783 (50.0)	1527 (52.7)
Living alone, *n* (%)	1396 (34.9)	1074 (38.4)	2042 (36.7)	1160 (40.1)
Income group, *n* (%)[a]				
Lowest	969 (24.5)	754 (27.5)	1,257 (22.9)	776 (27.3)
Second lowest	1014 (25.7)	729 (26.5)	1325 (24.1)	687 (24.2)
Second highest	1013 (25.6)	691 (25.2)	1397 (25.5)	654 (23.0)
Highest	954 (24.2)	572 (20.8)	1508 (27.5)	722 (25.4)
Unknown	50	48	74	57
Educational level, *n* (%)[a]				
Basic	1,328 (34.4)	974 (36.6)	1,735 (32.1)	952 (34.2)
High school or vocational	1741 (45.1)	1219 (45.8)	2388 (44.2)	1239 (44.5)
Higher	790 (20.5)	469 (17.6)	1276 (23.6)	596 (21.4)
Unknown	141	132	162	109
Ethnicity, *n* (%)				
Native Danish	3310 (82.8)	2253 (80.6)	4661 (83.8)	2350 (81.1)
Immigrants/Descendants	690 (17.2)	541 (19.4)	900 (16.2)	546 (18.9)
Requested by a GP, *n* (%)	3666 (91.7)	2516 (90.1)	4897 (88.1)	2484 (85.8)
Categories of first HbA1c, *n* (%)				
48-52 mmol/mol<br>6.5-6.9%	2805 (70.1)	1702 (60.9)	5045 (90.7)	2416 (83.4)
53-57 mmol/mol<br>7.0-7.4%	1195 (29.9)	1092 (39.1)	516 (9.3)	480 (16.6)
eGFR prior to first HbA1c, *n* (%)[a]				
90 ml/min per 1.73m^2	2064 (54.6)	1573 (60.0)	2555 (48.3)	1338 (49.2)
60-89 ml/min per 1.73m^2	1569 (41.5)	965 (36.8)	2398 (45.3)	1224 (45.0)
30-59 ml/min per 1.73m^2	148 (3.9)	85 (3.2)	340 (6.4)	157 (5.8)
Unknown	219	171	268	177
LDL-cholesterol prior to first HbA1c, *n* (%)[a]				
0-2.5 mmol/L	600 (21.5)	415 (21.6)	880 (22.4)	415 (20.5)
≥ 2.6 mmol/L	2197 (78.5)	1502 (78.4)	3049 (77.6)	1606 (79.5)
Unknown	1203	877	1632	875
Comorbidities, *n* (%)				
Atrial fibrillation	86 (2.1)	67 (2.4)	194 (3.5)	75 (2.6)
Hypertension	831 (20.8)	603 (21.6)	1006 (18.1)	535 (18.5)
COPD/Asthma	542 (13.6)	397 (14.2)	788 (14.2)	412 (14.2)
Chronic Kidney Disease	41 (1.0)	33 (1.2)	67 (1.2)	24 (0.8)
Cancer	183 (4.6)	120 (4.3)	313 (5.6)	144 (5.0)
Heart failure	65 (1.6)	50 (1.8)	127 (2.3)	70 (2.4)
Pharmacotherapy, *n* (%)[b]				

Insulin	58 (1.4)	121 (4.3)	-	-
Metformin	3937 (98.4)	2721 (97.4)	-	-
DPP-4i	77 (1.9)	113 (4.0)	-	-
SU	47 (1.2)	69 (2.5)	-	-
SGLT-2i	60 (1.5)	107 (3.8)	-	-
GLP-1RA	102 (2.5)	70 (2.5)	-	-
Statins	1888 (47.2)	1451 (51.9)	717 (12.9)	475 (16.4)
Antithrombotics	214 (5.3)	165 (5.9)	201 (3.6)	129 (4.5)
RASi	1182 (29.6)	847 (30.3)	883 (15.9)	471 (16.3)
Beta blockers	393 (9.8)	277 (9.9)	627 (11.3)	326 (11.3)
Thiazide	363 (9.1)	267 (9.6)	482 (8.7)	290 (10.0)
Ca channel blockers	516 (12.9)	369 (13.2)	697 (12.5)	364 (12.6)
Loop diuretics	226 (5.6)	189 (6.8)	340 (6.1)	196 (6.8)

[a]The percentages indicate the proportions among individuals with complete data
[b]The dashes indicate that none initiated GLDT in these groups
COPD: chronic obstructive pulmonary disease; DPP-4i: dipeptidyl peptidase-4 inhibitors; eGFR: estimated glomerular filtration rate; GLP-1RA: glucagon-like peptide-1 receptor agonists; GP: general practitioner; LDL: low-densitiy lipoprotein; RASi: renin-angiotensin system inhibitor; SGLT-2i: sodium glucose co-transporter 2 inhibitors; SU, sulfonylureas; T2D, type 2 diabetes.

ESM Table 5. Probabilities of initiating statins and RASi according to glucose-lowering drug treatment and initial glycaemic control, 365 days after index.

	On GLDT		Not on GLDT	
	Well-controlled (HbA1c < 48 mmol/mol)	Poorly controlled (HbA1c ≥ 48 mmol/mol)	Remission (HbA1c < 48 mmol/mol)	Persistent T2D (HbA1c ≥ 48 mmol/mol)
**Statin**				
1-year probabililty	56.4 (54.7, 58.2)	56.5 (54.3, 58.6)	20.1 (19.0, 21.1)	27.9 (26.4, 29.4)
**RASi**				
1-year probabililty	34.9 (33.3, 36.6)	36.8 (34.7, 38.9)	20.0 (19.0, 21.1)	22.8 (21.4, 24.2)

Index denotes 180 days after first measured HbA1c ≥ 48 mmol/mol. Probabilities are presented as percentages with 95% confidence intervals in parentheses. GLDT, glucose-lowering drug treatment; RASi, renin-angiotensin system inhibitor; T2D, type 2 diabetes

ESM Table 6. Level of LDL cholesterol according glucose-lowering drug treatment and initial glycaemic control, 365 days after index.

Most recent LDL cholesterol level 365 days after index	On GLDT		Not on GLDT	
	Well-controlled (HbA1c < 48 mmol/mol) (*n* = 3176)	Poorly controlled (HbA1c ≥ 48 mmol/mol) (*n* = 2092)	Remission (HbA1c < 48 mmol/mol) (*n* = 5446)	Persistent T2D (HbA1c ≥ 48 mmol/mol) (*n* = 3507)
**No. of patients with available LDL cholesterol (% of total)**	2133 (67.2)	1341 (64.1)	2853 (52.4)	1923 (54.8)
0-1.7 mmol/L (% of patients with available LDL cholesterol)	573 (26.9)	348 (26.0)	316 (11.1)	239 (12.4)
1.8-2.5 mmol/L (% of patients with available LDL cholesterol)	692 (32.4)	466 (34.8)	699 (24.5)	526 (27.4)
≥ 2.6 mmol/L (% of patients with available LDL cholesterol)	868 (40.7)	527 (39.3)	1838 (64.4)	1158 (60.2)
Median (mmol/L) (IQR)	2.3 [1.7, 3.0]	2.3 [1.7, 3.0]	2.9 [2.3, 3.5]	2.8 [2.2, 3.4]
**No. of patients with unknown level of LDL cholesterol (% of total)**	727 (22.9)	498 (23.8)	2056 (37.8)	1211 (34.5)
**No. of deaths, censoring, or follow-up less than 365 days (% of total)**	316 (9.9)	253 (12.1)	537 (9.9)	373 (10.6)

GLDT, glucose-lowering drug treatment; LDL: low-density lipoprotein; T2D, type 2 diabetes

ESM Figure 1. Distribution of glucose-lowering drug treatment and most recent HbA1c level at each time point up to 180 days after first measured HbA1c ≥ 48 mmol/mol.

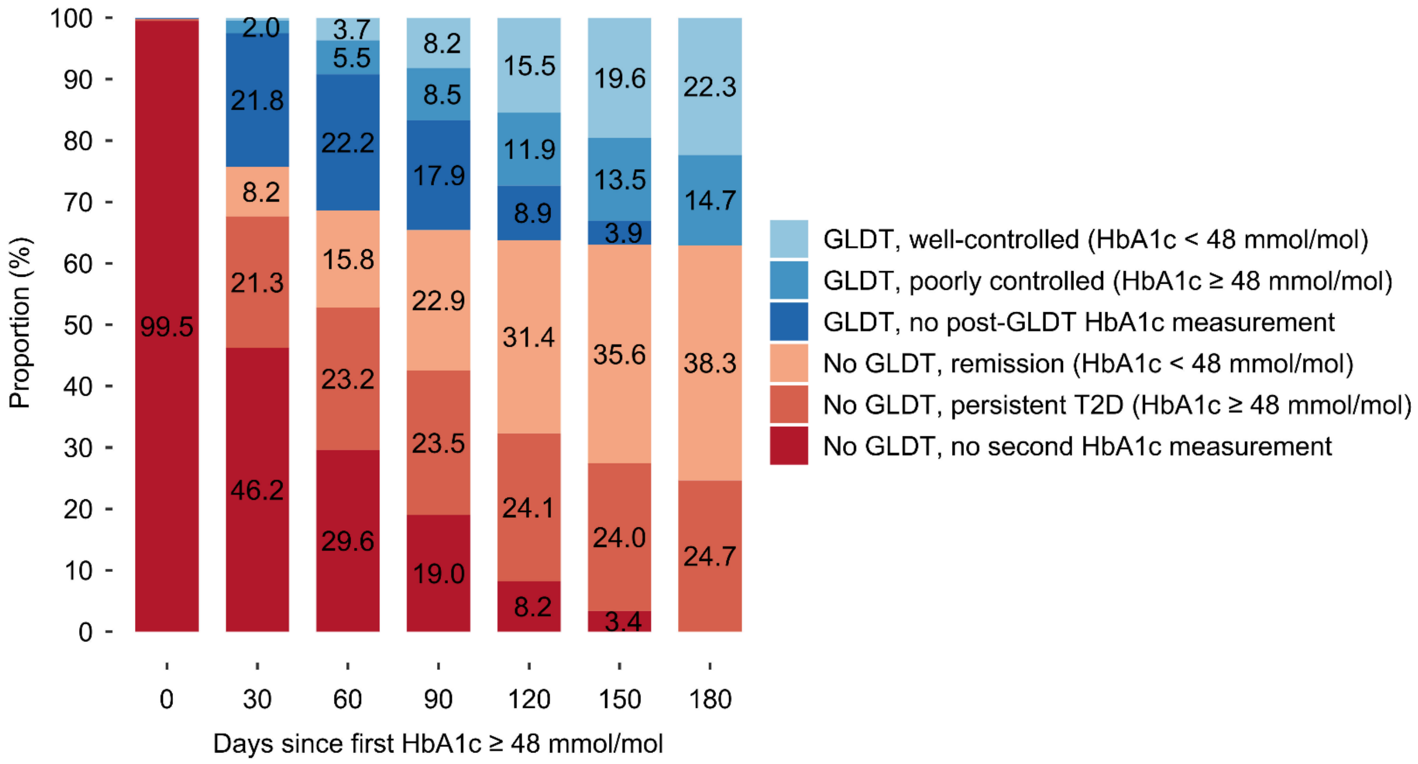

GLDT, glucose-lowering drug treatment; T2D, type 2 diabetes

ESM Figure 2. Probability of initiating statins (a) and RASi (b) according to glucose-lowering drug treatment and glycaemic control, 180 days after first measured HbA1c ≥ 48 mmol/mol, among men, stratified by age.

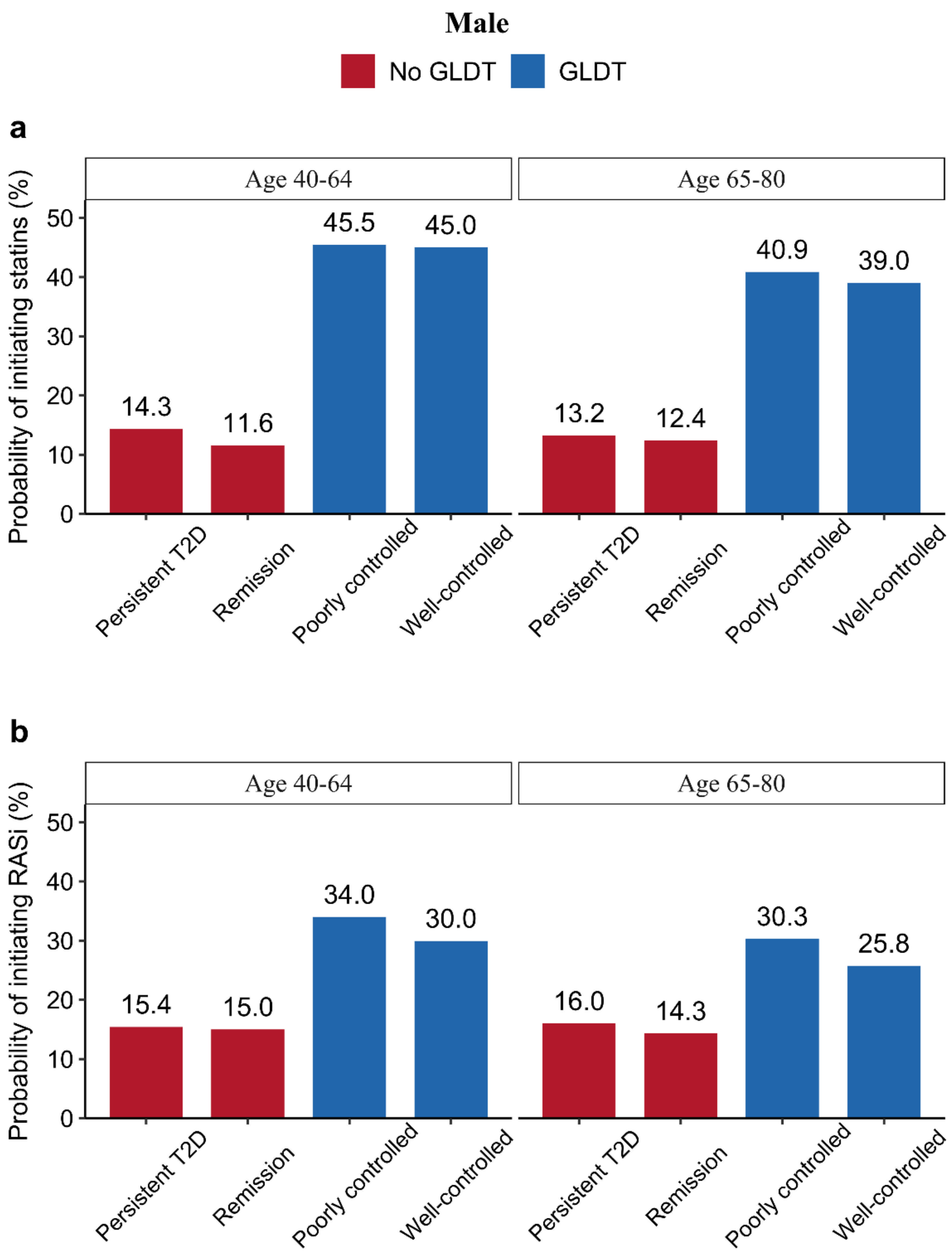

GLDT, glucose-lowering drug treatment; RASi, renin-angiotensin system inhibitor; T2D, type 2 diabetes.

ESM Figure 3. Probability of initiating statins (a) and RASi (b) according to glucose-lowering drug treatment and glycaemic control, 180 days after first measured HbA1c ≥ 48 mmol/mol, among women, stratified by age.

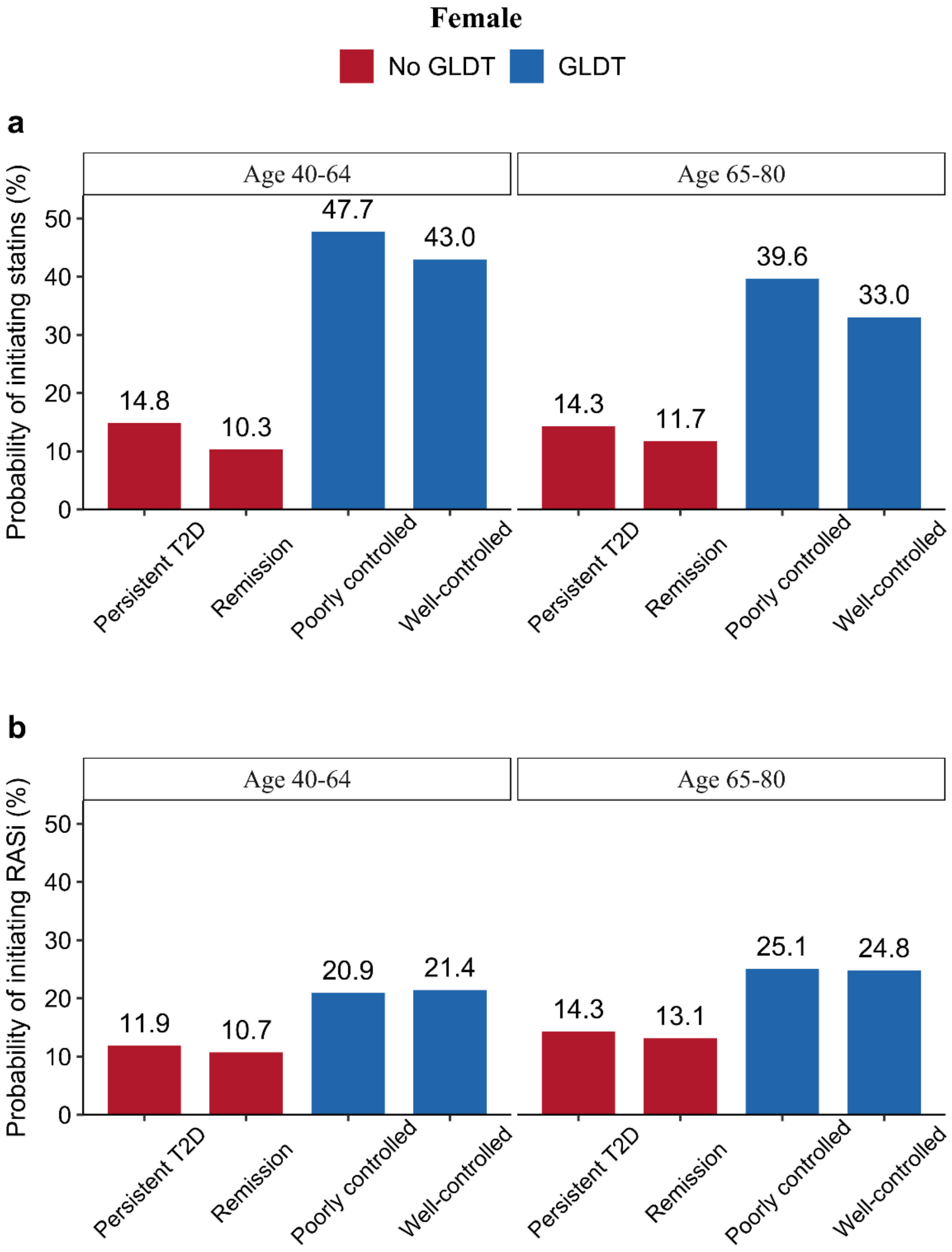

GLDT, glucose-lowering drug treatment; RASi, renin-angiotensin system inhibitor; T2D, type 2 diabetes.

ESM Figure 4. Probabilities of initiating statins (a) and RASi (b) within one year after index date according to glucose-lowering drug treatment and initial glycaemic control.

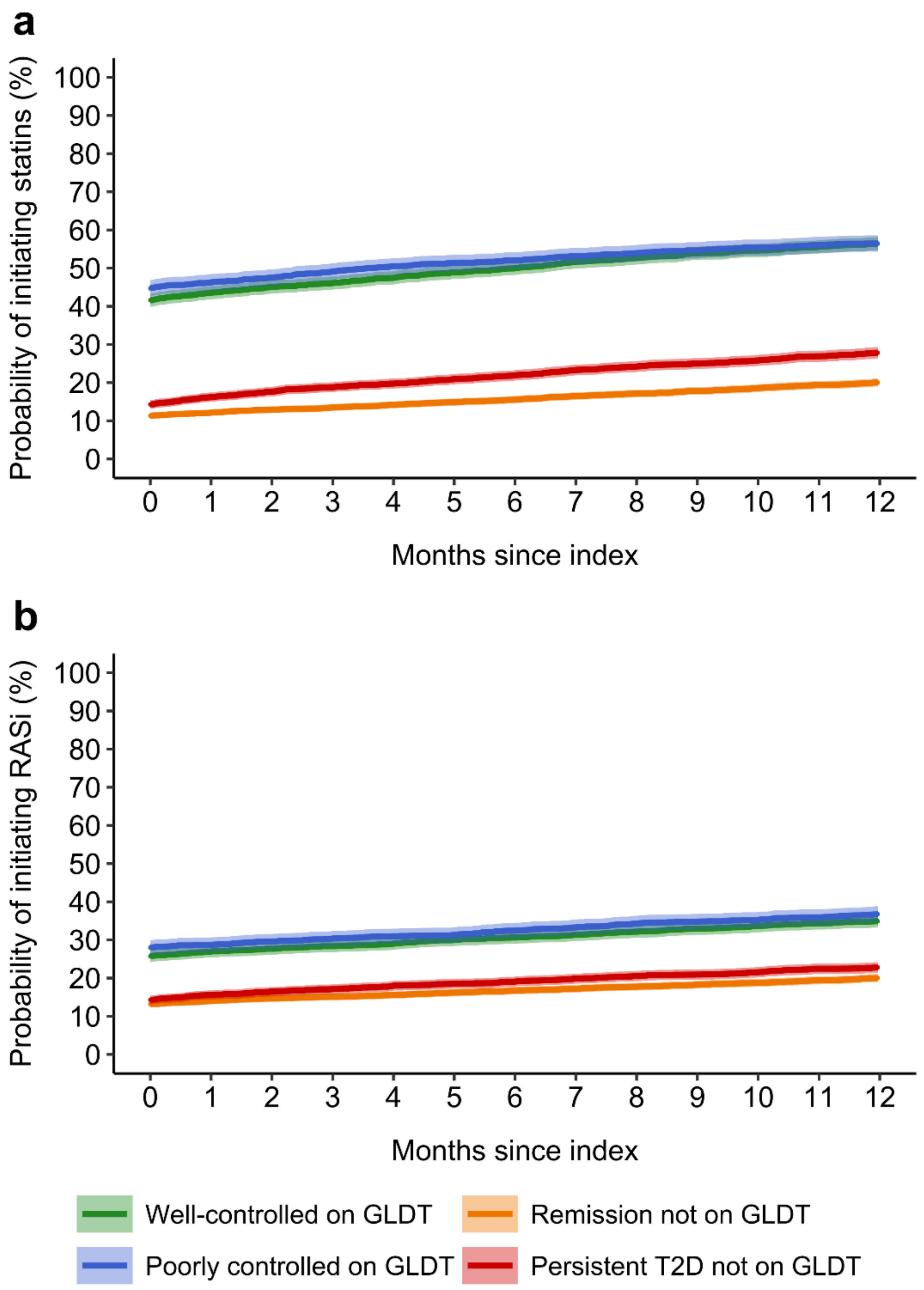

Index denotes 180 days after first measured HbA1c ≥ 48 mmol/mol. GLDT, glucose-lowering drug treatment; RASi, renin-angiotensin system inhibitor; T2D, type 2 diabetes.

ESM Figure 5. Standardised absolute 5 year risk of MACE according to glucose-lowering drug treatment and glycaemic control, 180 days after first measured HbA1c ≥ 48 mmol/mol, stratified by age and sex.

Glycaemic control & GLDT	Male: Standardised 5 year risk (%) (95% CI)	Male: Risk difference (%) (95% CI)	Female: Standardised 5 year risk (%) (95% CI)	Female: Risk difference (%) [95% CI]
**Age 40−64**				
Persistent T2D not on GLDT	10.8 (8.7, 12.8)	5.0 (2.5, 7.6)	7.2 (5.5, 9.2)	4.2 (1.9, 6.3)
Remission not on GLDT	8.4 (7.0, 9.9)	2.7 (0.6, 4.7)	7.3 (5.8, 9.0)	4.3 (2.2, 6.3)
Poorly controlled on GLDT	7.6 (5.7, 10.1)	1.9 (−0.5, 5.0)	6.4 (4.2, 8.7)	3.4 (0.8, 5.8)
Well−controlled on GLDT	5.7 (4.1, 7.4)	Reference	3.0 (1.9, 4.5)	Reference
**Age 65−80**				
Persistent T2D not on GLDT	25.5 (21.9, 29.3)	3.9 (−1.9, 9.6)	19.6 (15.7, 23.5)	−1.1 (−7.0, 4.3)
Remission not on GLDT	22.6 (19.3, 25.8)	1.0 (−4.6, 5.9)	19.4 (16.6, 22.4)	−1.2 (−6.5, 3.4)
Poorly controlled on GLDT	25.2 (20.1, 30.9)	3.6 (−2.9, 10.5)	29.7 (23.8, 36.1)	9.1 (2.0, 16.5)
Well−controlled on GLDT	21.6 (17.6, 26.0)	Reference	20.6 (16.9, 25.0)	Reference

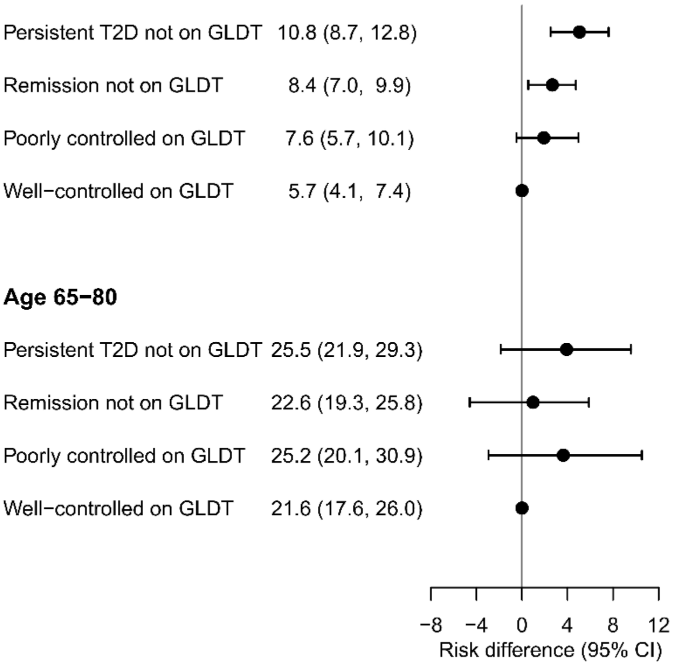

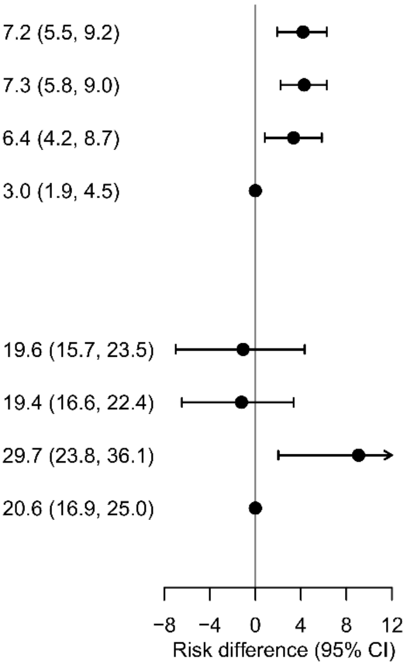

Standardised to the distribution of all included patients with respect to the distribution of residual age, cohabitation status, ethnicity, income, type of requester (general practitioner or other), first measured HbA1c level, estimated glomerular filtration rate, comorbidities, and after setting the use of statin and RASi as observed for each exposure group for all patients.
GLDT, glucose-lowering drug treatment; MACE, major adverse cardiovascular event consisting of stroke, myocardial infarction, or all-cause death; RASi, renin-angiotensin system inhibitor; T2D, type 2 diabetes

ESM Figure 6. Expected absolute reduction of standardised 5 year risk of MACE if each exposure group had the same probability of receiving statins and RASi as the well-controlled group on glucose-lowering drug treatment, stratified by sex and age.

Sex	Age group	Glycaemic control & GLDT	Observed risk (%)	Risk under intervention (%)	Reduction of risk under intervention (%) (95% CI)
Male	40−64 years	Persistent T2D not on GLDT	10.8	8.8	1.9 (0.2, 3.6)
		Remission not on GLDT	8.4	7.1	1.3 (−0.1, 2.5)
		Poorly controlled on GLDT	7.6	7.5	0.1 (−0.3, 0.7)
		Well−controlled on GLDT	5.7	−	−
Male	65−80 years	Persistent T2D not on GLDT	25.5	22.3	3.3 (0.4, 6.1)
		Remission not on GLDT	22.6	20.2	2.4 (−0.1, 4.8)
		Poorly controlled on GLDT	25.2	25.4	−0.2 (−1.3, 0.7)
		Well−controlled on GLDT	21.6	−	−
Female	40−64 years	Persistent T2D not on GLDT	7.2	6.1	1.1 (−0.2, 2.3)
		Remission not on GLDT	7.3	6.7	0.6 (−0.5, 1.7)
		Poorly controlled on GLDT	6.4	6.4	0.0 (−0.6, 0.4)
		Well−controlled on GLDT	3.0	−	−
Female	65−80 years	Persistent T2D not on GLDT	19.6	16.3	3.3 (1.6, 5.3)
		Remission not on GLDT	19.4	18.6	0.8 (−0.9, 2.4)
		Poorly controlled on GLDT	29.7	30.1	−0.4 (−2.5, 1.4)
		Well−controlled on GLDT	20.6	−	−

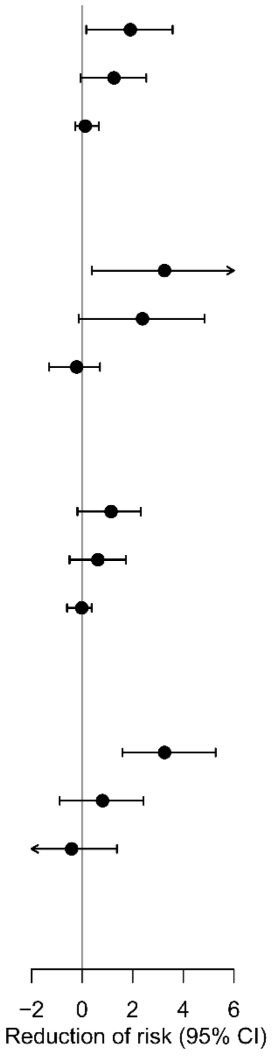

GLDT, glucose-lowering drug treatment; MACE, major adverse cardiovascular event consisting of stroke, myocardial infarction or all-cause death; RASi, renin-angiotensin system inhibitor; T2D, type 2 diabetes

ESM Figure 7. Standardised absolute 5 year risk of first-time MACE according to initial glucose-lowering drug treatment and glycaemic control, 365 days after first measured HbA1c ≥ 48 mmol/mol.

Glycaemic control & GLDT	Standardised 5 year risk (%) (95% CI)	Risk difference (%) (95% CI)
Persistent T2D not on GLDT	13.9 (12.4, 15.3)	3.5 (1.8, 5.2)
Remission not on GLDT	12.4 (11.4, 13.4)	2.0 (0.5, 3.4)
Poorly controlled on GLDT	12.3 (10.8, 13.7)	1.8 (0.0, 3.5)
Well−controlled on GLDT	10.5 (9.3, 11.7)	Reference

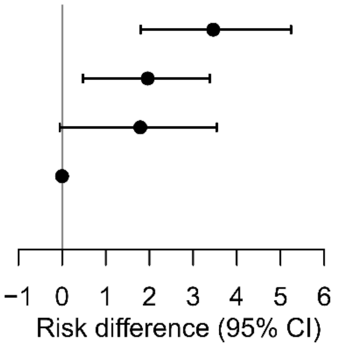

Standardised to the distribution of all included patients with respect to the distribution of age, sex, cohabitation status, ethnicity, income, type of requester (general practitioner or other), first measured HbA1c level, estimated glomerular filtration rate, comorbidities, and after setting the use of statin and RASi as observed for each exposure group for all patients.

GLDT, glucose-lowering drug treatment; MACE, major adverse cardiovascular event consisting of stroke, myocardial infarction, or all-cause death; RASi, renin-angiotensin system inhibitor; T2D, type 2 diabetes

ESM Figure 8. Probability of initiating statins (a) and RASi (b) according to glucose-lowering drug treatment and initial glycaemic control, 365 days after first measured HbA1c ≥ 48 mmol/mol.

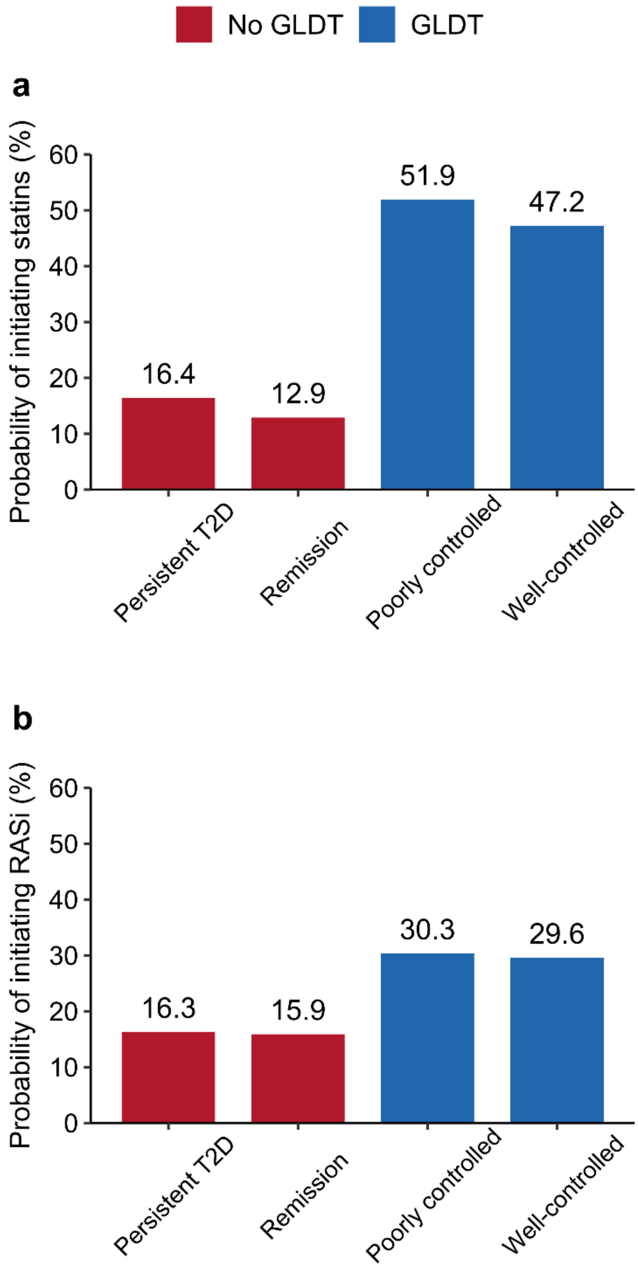

GLDT, glucose-lowering drug treatment; RASi, renin-angiotensin system inhibitor; T2D, type 2 diabetes

ESM Figure 9. Expected absolute reduction of standardised 5 year risk of MACE if each exposure group had the same probability of receiving statins and RASi as the well-controlled group on glucose-lowering drug treatment, 365 days after first measured HbA1c ≥ 48 mmol/mol.

Glycaemic control & GLDT	Observed risk (%)	Risk under intervention (%)	Reduction of risk under intervention (%) (95% CI)
Persistent T2D not on GLDT	13.9	11.4	2.6 (1.6, 3.5)
Remission not on GLDT	12.4	11.1	1.4 (0.6, 2.1)
Poorly controlled on GLDT	12.3	12.3	−0.1 (−0.4, 0.2)
Well−controlled on GLDT	10.5	-	-

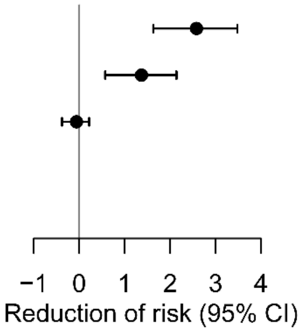

GLDT, glucose-lowering drug treatment; MACE, major adverse cardiovascular event consisting of stroke, myocardial infarction or all-cause death; RASi, renin-angiotensin system inhibitor; T2D, type 2 diabetes.
